# Supplementary material for: Association between oral microbiome and seven types of cancers in East Asian population: a two-sample Mendelian randomization analysis
Source: Front Mol Biosci. 2023 Nov 21;10:1327893. doi: 10.3389/fmolb.2023.1327893 (PMC10702768; doi:10.3389/fmolb.2023.1327893)
Supplement: Supplementary file 1 [file DataSheet1.ZIP › Supplementary Materials/MR plots for tongue/tongue═╝/Gastric cancer/pheno.952_to_gastric cancer_forest.pdf]

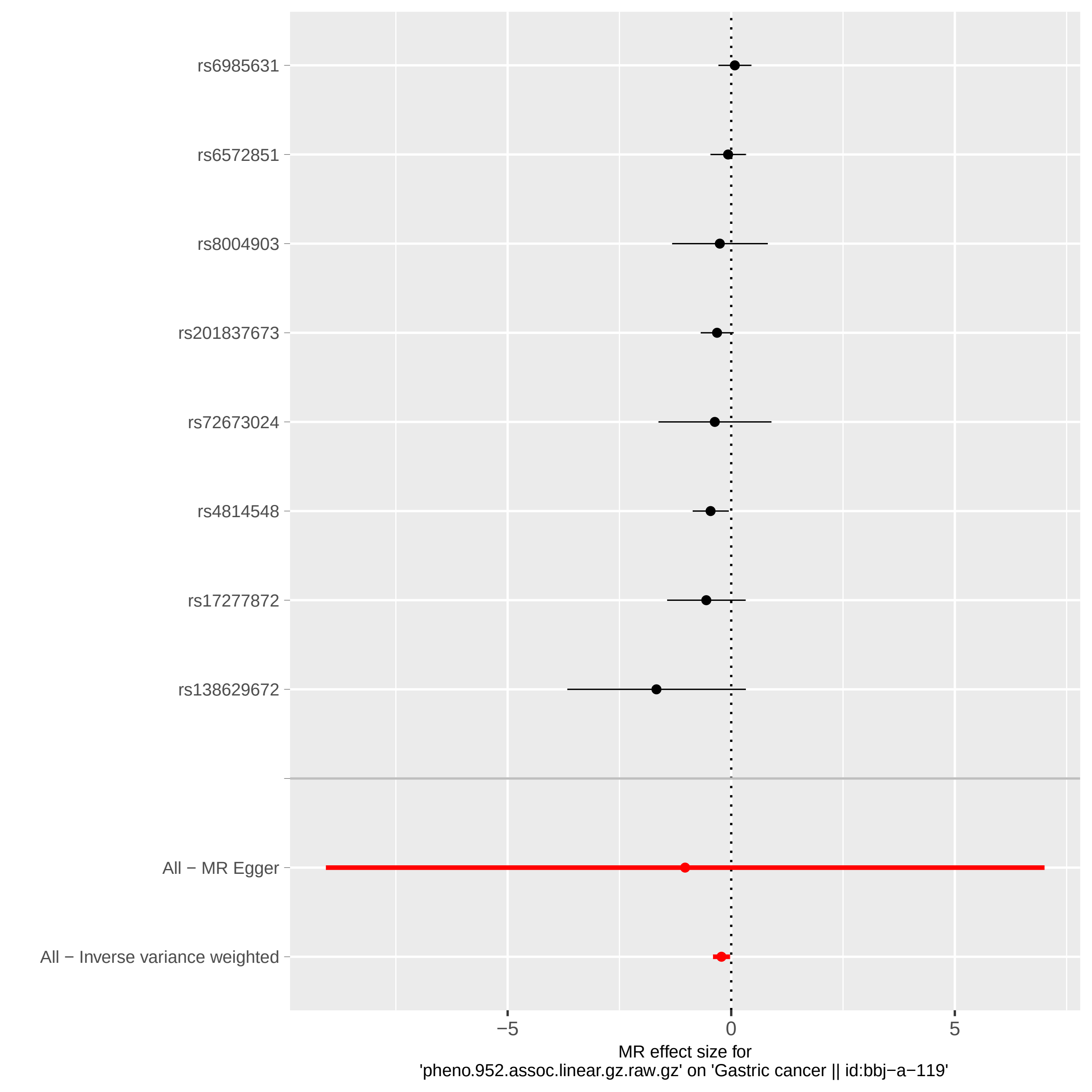
rs6985631
rs6572851
rs8004903
rs201837673
rs72673024
rs4814548
rs17277872
rs138629672
All − MR Egger
All − Inverse variance weighted
−5
0
5
MR effect size for
'pheno.952.assoc.linear.gz.raw.gz' on 'Gastric cancer || id:bbj−a−119'
